# Maternal and Fetal Outcomes of Pregnancies in Wilson’s Disease: A Single-Centre Real-Life Experience

**DOI:** 10.5152/tjg.2025.25448

**Published:** 2025-10-22

**Authors:** Kadir Demir, Aslı Çifcibaşı Örmeci, Bilger Çavuş, Zülal İstemihan, Sezen Genç, Alp Atasoy, Filiz Akyüz, Fatih Beşışık, Sabahattin Kaymakoğlu

**Affiliations:** Division of Gastroenterohepatology, Department of Internal Medicine, İstanbul University İstanbul Faculty of Medicine, İstanbul, Türkiye

**Keywords:** Chelator agents, congenital abnormalities, pregnancy, Wilson’s disease

## Abstract

**Background/Aims:**

This study aims to investigate the fetal outcomes and pregnancy course in patients with Wilson’s disease (WD).

**Materials and Methods:**

A total of 54 female patients with a diagnosis of WD and a history of pregnancy between 1985 and 2021 were retrospectively examined in a specifically WD outpatient clinic in the İstanbul Faculty of Medicine.

**Results:**

A total of 131 pregnancies in 54 patients were examined. The mean age at diagnosis of WD was 22.1 ± 10.8 years. The mean follow-up period of patients diagnosed with WD was 87 ± 80 months. Forty (74.1%) patients were diagnosed with hepatic, 5 (9.3%) neurological, and 9 (16.6%) with mixed involvement WD. The liver was cirrhotic in 68 (51.9%) pregnancies. Ninety-eight (74.8%) pregnancies resulted in live birth, 30 spontaneous abortions (22.9%), and 3 (2.3%) stillbirths. Pregnancies diagnosed with mixed pattern WD had a lower live birth rate than those diagnosed with WD with isolated hepatic or neurological involvement (*P* < .001 and *P* = .004, respectively). While the spontaneous abortion rate decreases with WD treatment, the rate of live birth, stillbirth, premature birth, and low birth weight did not change in the total group. During pregnancy, patients used D-penicillamine, trientine, and/or zinc.

**Conclusion:**

With careful monitoring and management by medical professionals, pregnant women diagnosed with WD can still achieve successful pregnancies and improve their quality of life.

**Main Points**

- Data on the course and management of pregnancy in Wilson’s disease (WD) are limited.
- Pregnancy in WD can be challenging due to increased risks of miscarriage, preterm birth, and perinatal mortality.
- Close monitoring of liver function and continuation of copper-chelating therapy are essential to minimize the risk of complications during pregnancy.

## Introduction

Wilson’s disease (WD) is an autosomal recessive disorder of copper metabolism caused by mutations in the *ATP7B* gene with a prevalence of 1 in 30 000.^1^ Mutations in this gene disrupt copper excretion from the liver into the biliary system, leading to copper accumulation in the liver, brain, corneal tissue, kidneys, and other organs. This accumulation subsequently leads to complications, primarily affecting the liver and nervous system. Wilson’s disease is mostly diagnosed between the ages of 5 and 35 years, while the mean onset of hepatic and neurological symptoms is between the ages of 9 and 13 years and 15 and 21 years, respectively.^2^

The relationship between WD and pregnancy is of significant importance as the onset of the disease typically coincides with the reproductive period and earlier stages of life.^3^ Due to the relatively low prevalence of WD and the consequently limited sample size, comprehensive studies on the management of WD during pregnancy are unfortunately very rare in the literature. Many recommendations in guidelines remain at the level of expert opinion due to the limited number of studies.

However, in women with known WD, serum copper and ceruloplasmin levels can rise during pregnancy without treatment, leading to a flare in symptoms.

During pregnancy, patients should be monitored closely for hepatic and neurological symptoms. The general recommendation, based on low-quality evidence, supports the maintenance of WD treatment during pregnancy. The potential teratogenic effect of chelating agents and maternal copper deficiency that may develop in association with treatment may be risk factors for the fetus. Overtreatment with chelating agents can cause copper deficiency, and it is known that copper plays an important role in maternal and fetal health. Several studies indicated that deficiency in copper during pregnancy is associated with low birth weight (LBW) and preterm delivery.^4^

D-penicillamine and trientine have known teratogenic effects, but limited data demonstrate safe use during pregnancy, with a recommended dose reduction of approximately 50% to start during the first trimester and continued through the pregnancy to reduce possible adverse effects on the fetus and to support postpartum wound healing in the mother. While it is accepted that chelator agents can be continued by decreasing during pregnancy in the last guideline of the American Association for the Study of Liver Diseases 2022, it is recommended that chelator treatment can be discontinued during pregnancy and pregnant women can be monitored only with zinc treatment.^5^ However, before making such a change, it should be ensured that the patient’s clinical condition is good before pregnancy.

Feeding the baby with breast milk is another problem that needs to be clarified in those receiving chelator treatment. There is limited information regarding the passage of oral chelators into breast milk. Penicillamine passes into breast milk and may be harmful to the baby. The amount of copper in breast milk may be insufficient to meet the needs of the newborn. Zinc bioavailability in breast milk is quite good, but information on the effect of pharmacological doses of zinc on breast milk concentrations is limited.

In light of all these data, there are publications in the literature in the form of case series about the pregnancy and fetal outcomes of WD patients. The largest-scale analysis in the literature was conducted on 282 pregnancies in 136 WD patients, and both pregnancy results and fetal outcomes were reported.^6^

This study describes a single-center case series examining pregnancy and fetal outcomes in patients with WD.

## Materials and Methods

A total of 54 female patients with a diagnosis of WD and a history of pregnancy between 1985 and 2021 were retrospectively examined in a specifically WD outpatient clinic in the İstanbul Faculty of Medicine. Patient information was obtained from the hospital registration system. The study was planned retrospectively. Wilson’s disease was diagnosed according to the Leipzig criteria. Those with a Leipzig score ≥ 4 were considered to have WD (Table 1).^5^

**Figure 1.**
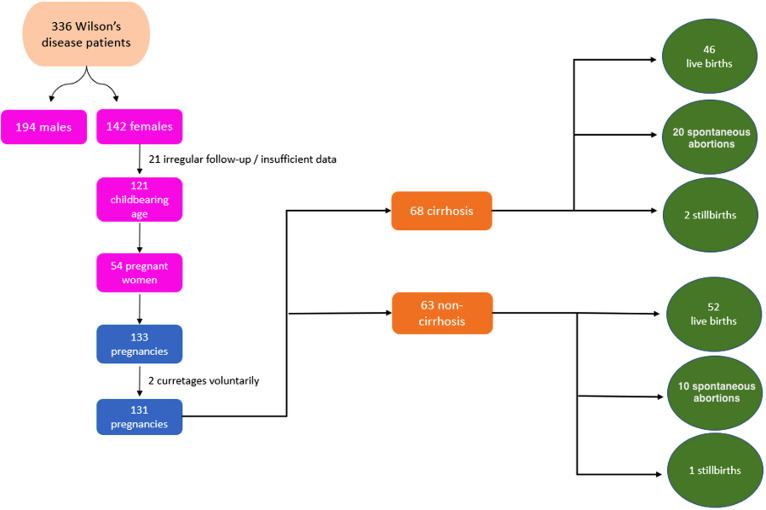
Flowchart of the study.

Of the 336 WD patients (142, 42.3% female) followed at the center, 121 (38.3%) were women of childbearing age. A total of 133 pregnancies occurred in 54 of these. Two of these pregnancies were terminated by curettage voluntarily, except for medical necessity. A total of 131 pregnancies were evaluated (Figure 1).

Data collected included: age at diagnosis, clinical presentation (i.e., asymptomatic, hepatic, neurologic, and/or neuropsychiatric), pharmacological treatment for WD (i.e., zinc, D-penicillamine, trientine, or combined) prior to and during pregnancy, duration of pharmacological treatment before pregnancy, characteristics of all pregnancies and their results (total number of pregnancies, number of spontaneous abortions, number of stillbirths, number of live births, whether treatment was received for WD during pregnancy, week of gestation, weights of the babies born), maternal complications during pregnancy, rate of spontaneous abortions, and birth defects were investigated with respect to medical treatment during pregnancy.

Those who got pregnant before WD diagnosis and pregnancies before 2000 were evaluated retrospectively. The follow-up of the patients since 2000 was as follows. Patients who became pregnant after the diagnosis of WD were informed before pregnancy, and in case of pregnancy, they were advised to reduce the chelator treatment to a twice-daily dose and immediately apply to the outpatient clinic. After confirmation of pregnancy, 24-hour urine copper, serum copper levels, serum ceruloplasmin levels, liver enzymes, and other biochemical tests were evaluated. These examinations were repeated after diagnosis every 3 months, and the last time at the eigth month of the pregnancy. The type of delivery was determined by gynecologists. During the lactation period, the dose was continued as a twice-daily dose. All pregnant women were advised to stop lactation at the sixth month, but some pregnant women extended the duration of lactation at their request.

Ethical committee approval was received from the Ethics Committee of the İstanbul University, Istanbul Faculty of Medicine (Approval No.: 181150; Date: April 26, 2024). The requirement for written informed consent was waived due to the study’s retrospective nature.

### Statistical Analysis

Quantitative data are described as mean, SD, and median (interquartile range [IQR]). Sample size (n) and percentage (%) were used to describe the quantitative data. The chi-square test was calculated to determine the relationships between categorical variables. All statistical analyses were performed using the IBM SPSS 22.0 program (IBM SPSS Corp.; Armonk, NY, USA), and *P* < .05 (2-tailed) was considered statistically significant.

## Results

There were 54 WD patients in the study. Forty (74.1%) patients were diagnosed with hepatic involvement, 5 (9.3%) with neurological involvement, and 9 (16.7%) with mixed involvement WD. The mean age at diagnosis of WD was 22.1 ± 10.8 years [median 19, IQR (25-75) (13.6-30.8) years]. The mean follow-up duration was 87 ± 80 months (6-316 months) [median 70 months, IQR (25-75) (11.5-141) months]. Of these 54 pregnant women, 19 (34.5%) had consanguineous marriages, and 20 (36.4%) had a family history of WD.

A total of 133 pregnancies occurred in 54 women. Since 2 pregnancies were terminated by curettage, a total of 131 pregnancies in 54 patients were examined. Sixty-three (48.1%) of these pregnancies occurred before the diagnosis of WD, and 68 (51.9%) after the diagnosis of WD.

Ninety-eight (74.8%) pregnancies resulted in live birth. Thirty-three (25.2%) pregnancies could not be completed due to 30 spontaneous abortions (22.9%) and 3 (2.3%) stillbirths. Forty-four (83%) of 53 pregnancies with known gestational age were born at normal gestational age (38-42 weeks), and 9 (17%) pregnancies resulted in preterm birth (<38 weeks). Of the 61 babies with known birth weight, 54 (88.5%) were of normal weight (2500-4000 grams), 6 (9.9%) were underweight (<2500 grams), and 1 (1.6%) was overweight (>4000 grams). The distribution of pregnancy and its outcomes before and after WD diagnosis are given in Table 2.

One patient with WD also had antiphospholipid syndrome. The patient with antiphospholipid syndrome had 11 pregnancies, including 7 miscarriages, before the diagnosis of WD. The patient had 2 miscarriages and 2 live births after treatment.

Wilson’s disease treatment was given during pregnancy in 62 of 68 pregnancies (91.2%) that occurred after the diagnosis of WD. Six (8.8%) patients voluntarily discontinued their treatment and followed up without treatment because of the anxiety of the drug’s side effects. Only zinc was used in 4 pregnancies, only penicillamine in 19, only trientine in 4, zinc and penicillamine in 33, and zinc and trientine in 2. When the treated patient groups were compared with each other, the rate of birth with LBW was significantly higher in patients receiving trientine alone (*P* = .001); there were no other differences in live births, miscarriages, stillbirths, or gestational age by treatment (*P* > .05).

Seventy-nine (60.3%) pregnancies occurred under the diagnosis of WD with hepatic involvement. Twelve (9.2%) were diagnosed with WD with neurological involvement, and 40 (30.5%) pregnancies were diagnosed with WD with mixed involvement.

Whether the pregnancy was before the diagnosis of WD or after the diagnosis of WD and receiving WD treatment did not affect the completion of the pregnancy (live birth) (69.8% pregnancy before diagnosis vs. 82.3% pregnancy after diagnosis, *P* = .15) (Table 3). The comparison was made with 63 pregnancies that developed before diagnosis and 62 pregnancies that developed after diagnosis and continued treatment. In pregnancies that occurred after the diagnosis of WD, whether or not receiving treatment did not affect the completion of the pregnancy (live birth) (50% in not receiving treatment vs. 82.3% in receiving treatment, *P* = .06) (Table 4).

When the group of 6 pregnancies that stopped treatment after diagnosis was compared with the 62 pregnancies that continued treatment, it was seen that the rate of spontaneous abortion was significantly higher in the group that stopped treatment (50% in the group that discontinued treatment vs 12.9% in the group that continued treatment, *P* = .01) (Table 4).

**Figure 2.**
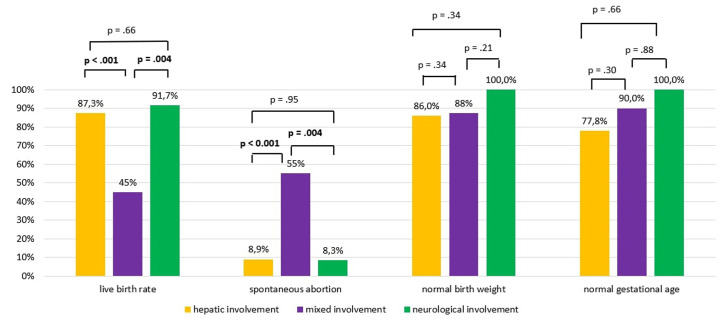
Rates of live birth, spontaneous abortion, birth at normal weight, and birth at normal gestational age according to clinical forms of Wilson’s disease.

Pregnancies diagnosed with mixed pattern WD had a lower live birth rate than those diagnosed with WD with isolated hepatic or neurological involvement (45% in mixed involvement vs 87.3% in hepatic involvement, *P* < .001, 45% in mixed involvement vs 91.7% in neurological involvement, *P* = .004, respectively) (Figure 2).

The spontaneous abortion rate among all pregnancies in the study group is 22.9%. The spontaneous abortion rate was significantly higher in pregnancies that occurred before the diagnosis of WD (30.1% in pregnancies that occurred before the diagnosis of WD vs 12.9 in pregnancies that occurred after the diagnosis of WD and received WD treatment, *P* = 0.03) (Table 3). The spontaneous abortion rate decreases with WD treatment. The spontaneous abortion rate in pregnancies diagnosed with WD with mixed involvement was significantly higher than both those with isolated hepatic involvement and those with isolated neurological involvement (55% in mixed pattern WD vs. 8.9% in hepatic pattern WD, and 55% in mixed pattern WD vs. 8.3% in neurological pattern WD; *P* < .001, and *P* = .004, respectively). Those with isolated hepatic and neurological involvement were not different from each other (*P* = .95) (Figure 2).

The gestational age of 10 of those with mixed involvement was known; 9 (90%) of them were born at normal gestational age, and 1 was born prematurely. The gestational age of 36 of those with hepatic involvement was known; 28 (77.8%) of them were born at normal gestational age, and 8 (22.2%) of them were born prematurely. The gestational age of 7 of the neurological patients was known. All were born with normal gestational age. The birth rate at normal gestational age was not different between the groups (*P* > .05) (Figure 2).

An increase in aminotransferases was observed in one of the patients under treatment, and an increase in neurological findings was observed in the other. However, this situation was not at a level that required a change in treatment. The patients were monitored without complications.

Of 131 pregnancies, birth defects occurred in 3 (3.1%) babies. One baby was born with anencephaly, another with an abdominal wall defect, and the last 1 had renal agenesis.

Galactosemia was detected in a baby during postnatal follow-up. He is now living a healthy life.

The liver was cirrhotic in 68 pregnancies (all the cases had Child A cirrhosis). Of these pregnancies, 46 (67.6%) resulted in live birth, 20 (29.4%) in spontaneous abortion, and 2 in stillbirth. There was no difference in live birth and spontaneous abortion rates between cirrhotic and non-cirrhotic pregnancies (67.6% live birth in cirrhotic pregnancies vs. 82.5% in non-cirrhotic pregnancies, and 29.4% spontaneous abortion in cirrhotic pregnancies vs. 15.9% in non-cirrhotic pregnancies; *P* = .05 and *P* = .06, respectively). The rate of normal birth weight was not different between cirrhotic and non-cirrhotic pregnancies (94.7% without cirrhosis vs. 78.3% with cirrhosis, *P* = .09), but the LBW rate was significantly higher in cirrhotic patients (2.6% without cirrhosis vs. 21.7% with cirrhosis, *P* = .02) (Table 5).

When those with known gestational age were examined, no relationship was found between being born at normal gestational age or preterm and pregnancy occurring before or after the diagnosis of WD, whether or not receiving treatment, and the clinical phenotype of WD (*P* > .05).

When those with known birth weight were examined, no relationship was found between being born with normal or low weight and pregnancy occurring before or after the diagnosis of WD, whether or not receiving treatment, and the clinical phenotype of WD (*P* > .05).

## Discussion

Wilson’s disease is an autosomal recessive copper metabolism disorder.^7^ A significant portion of female patients with WD are of reproductive age. There is very little information about the course of pregnancy in patients diagnosed with WD. Some patients are unaware that they have been diagnosed with WD when they become pregnant. Approximately half of the pregnancies in this study occurred before the patients were diagnosed with WD.

The rate of spontaneous abortion may be 10%-20% in the general population.^8^^,^^9^ There are case series regarding the miscarriage rate in patients with WD. In the study performed by Pfeiffenberger et al^6^ in 2018, the largest group of WD patients and pregnancies (136 women and 282 pregnancies) in the literature, there was a high rate of spontaneous abortions (40%) in untreated WD patients and those who stopped anti-copper treatment during pregnancy compared with the general population (10%-20%). In this study, spontaneous abortion occurred in 19 (30.1%) of 63 pregnancies that occurred before WD was diagnosed, and in 12.9% of 62 pregnancies that occurred after WD was diagnosed and received WD treatment. The spontaneous abortion rate decreased significantly with WD treatment. Likewise, when comparing the 6 pregnancies (50%) that stopped their treatment after pregnancy and the 62 pregnancies (12.9%) that continued their treatment after pregnancy, it was seen that the rate of spontaneous abortion was significantly lower in the group that continued their treatment. It is thought that the significant difference in the comparison despite the small number in the group that did not continue treatment shows the importance of treatment during pregnancy. It was found that the live birth rate was not affected by discontinuation of WD treatment, but this may be related to the small number of groups compared. Here, 6 patients who stopped their treatment after diagnosis were compared with 62 patients who continued their treatment, and the statistics were not significant due to the small number of the group that stopped treatment. In the same study,^5^ it was seen that most of the spontaneous abortions occurred in WD patients with hepatic and mixed involvement. In this study, isolated hepatic involvement did not affect the risk of spontaneous abortion, but in mixed involvement, the spontaneous abortion rate was significantly higher and the live birth rate was lower.

According to the literature, the following risk factors were identified at the time of conception for spontaneous abortion in WD: the presence of neurological symptoms (40% of abortions) and trends according to portal hypertension (29% of abortions) and liver cirrhosis (26% of abortions). The rate of spontaneous abortion is up to 30%-40% in patients with cirrhosis of any cause.^8^ Advanced chronic liver disease at presentation is associated with poor outcomes such as death and the need for liver transplantation. In this study, 20 (29.4%) of the 68 pregnancies in patients with WD-related cirrhosis resulted in spontaneous abortion, 2 (2.9%) in stillbirth, and 46 (67.6%) in live births. In this case series, all patients had Child A cirrhosis.

D-penicillamine has been successfully used in pregnancy, although there are reports of birth defects. In this study, the most commonly used chelator in treated pregnancy (n = 62) was D-penicillamine and zinc (n = 52, 83.9%, and n = 37, 59.7%, respectively). Some studies have shown D-penicillamine-associated congenital anomalies such as connective tissue disorders, low-set ears, and micrognathia.^10^^-^^12^ In this study, one of the babies born from 3 pregnancies followed by D-penicillamine had renal agenesis, anencephaly, and abdominal wall defect. These patients were evaluated for copper deficiency, and biochemical tests showed that there was no copper deficiency. When the literature was examined, no cases of D-penicillamine-related renal agenesis, anencephaly, and abdominal wall defect were found.

In the meta-analysis conducted by Tomasz Litwin and colleagues on the fetal and pregnancy outcomes of patients with WD, birth defects were found to be 4%.^13^ Similar to the literature, birth defects occurred in 3.1% of babies in the current data.

Galactosemia was detected after birth in a baby whose mother was under treatment. Hereditary galactosemia is an inherited carbohydrate metabolism disorder, and there is no relationship between the baby with galactosemia born to a mother with WD.^14^

In a meta-analysis, the rate of premature birth in pregnancies diagnosed with WD was found to be 2%.^15^ In this study, the premature birth rate was found to be 15.8% in the pregnancy group before diagnosis, and 17.6% in the pregnancy group after the diagnosis, which is quite high compared to the literature. In this study, treatment did not affect premature birth.

In a study conducted in Poland, the total birth rate below 2500 grams was found to be 10.9% in pregnant women who were and were not treated for WD.^16^ In this study, this rate was found to be 9.8%, which is consistent with the literature.

This study has some limitations. Its primary limitation is its single-center, retrospective nature. Furthermore, due to its retrospective nature, some patient data is incomplete due to the attempt to access patient data through a hospital record system search. However, given the relatively limited literature on this topic, it is believed that this study will contribute significantly to this topic and support future research.

In conclusion, pregnancy in women with WD can be problematic due to the high risk of miscarriage, premature birth, and perinatal mortality. However, with careful monitoring and management by medical professionals, WD pregnant women can still achieve successful pregnancies and improve their quality of life. The rate of spontaneous abortion is significantly low in patients monitored with treatment in WD. It can be said that treatment with a reduced dose of chelator agents and zinc is safe during pregnancy.

It is important to monitor liver function and provide treatment with copper-excretion medications during pregnancy in order to mitigate the risk of complications. More case series are needed to make decisions about better management in pregnant women diagnosed with WD.

## Figures and Tables

**Table 1.** The Leipzig Score for the Diagnosis of Wilson’s Disease

**Specific Clinical Features** Kayser–Fleischer rings (by slit-lamp examination) Present Absent	**Points** 2 0
Neuropsychiatric symptoms suggestive of WD (or typical features on brain MRI) Present Absent	2 0
Coombs-negative (nonimmune) hemolytic anemia (plus high serum copper) Present Absent	1 0
**Laboratory tests** 24-hour urinary copper excretion (in the absence of acute hepatitis) Normal 1-2× ULN >2× ULN Normal but >500 μg/day 1 day after challenge with 2 × 500 mg D-penicillamine	0 1 2 2
Liver copper quantitative Normal (ULN = 50 μg/g dry weight) Up to 5× ULN >5× ULN	−1 1 2
Rhodanine-positive hepatocytes (only if quantitative copper measurement not available) Absent Present	0 1
Serum ceruloplasmin (nephelometric assay, LLN = 20 mg/dL) Normal 10-20 <10	0 1 2
**Mutation analysis (ATP7B)** Disease-causing mutations on both chromosomes Disease-causing mutation on 1 chromosome No disease-causing mutation detected	4 1 0
**Assessment of the WD-diagnosis score** 4 or more: Diagnosis of Wilson’s disease highly likely 2-3: Diagnosis of Wilson’s disease probable, do more investigations 0-1: Diagnosis of Wilson’s disease unlikely	

ATP7B, ATPase copper transporting beta; LLN, lower limit of normal; MRI, magnetic resonance imaging; ULN, upper limit of normal; WD, Wilson’s disease.

**Table 2.** Distribution of Pregnancy and its Outcomes Before and After Wilson’s Disease Diagnosis

	Before Wilson’s Disease Diagnosis	After Wilson’s Disease Diagnosis	
Pregnancy number (n, %)	63 (48.1)	68 (51.9)	
WD involvement pattern Hepatic involvement (n, %) Neurological involvement (n, %) Mixed involvement (n, %)	35 (55.6) 5 (7.9) 23 (36.5)	44 (64.7) 7 (10.3) 17 (25)	
Cirrhosis (n, %)	40 (63.5)	28 (41.2)	.01
Live birth (n, %)	44 (69.8)	54 (79.4)	.28
Spontaneous abortion (n, %)	19 (30.1)	11 (16.2)	.09
Stillbirth (n, %)	0	3 (4.4)	.09
Premature birth (n = 53)^*^ (n, %) (19/34)	3 (15.8)	6 (17.6)	.86
Low birth weight (n = 61)^**^ (18/43)	1 (5.6)	6 (13.9)	.34
Receiving treatment (n, %)	–	62 (91.2)	
WD treatment distribution** (n = 62) Zinc (n) D-penicillamine (n) Trientine (n) Zinc and D-penicillamin (n) Zinc and trientine (n)	– – – – –	4 19 4 33 2	

WD, Wilson’s disease.

*Gestational age could be obtained in 53 pregnancies and birth weight in 61 pregnancies.

**Six patients voluntarily discontinued their treatment during pregnancy.

**Table 3.** Results According to the Time of Pregnancy

	Pregnancy Occurred Before WD Diagnosis (n = 63) (n, %)	Pregnancy Occurred After WD Diagnosis and Taking WD Treatment (n = 62) (n, %)	*P*
Live birth (n, %)	44 (69.8)	51 (82.3)	.15
Spontaneous abortion (n, %)	19 (30.1)	8 (12.9)	.03
Stillbirth (n, %)	0 (0)	3 (4.8)	.77
Premature birth [n = 53, (19/34) (n, %)]	3 (15.8)	6 (17.6)	.86
Low birth weight [n = 60, (18/42) (n, %)]	1 (5.6)	5 (11.9)	.45

WD, Wilson’s disease.

**Table 4.** Pregnancy Outcomes by Treatment Status

	Pregnancy Occurred After WD Diagnosis and Not Taking WD Treatment (n = 6) (n, %)	Pregnancy Occurred After WD Diagnosis and Taking WD Treatment (n = 62) (n, %)	*P*
Live birth (n, %)	3 (50)	51 (82.3)	.06
Spontaneous abortion (n, %)	3 (50)	8 (12.9)	.01
Stillbirth (n, %)	0 (0)	3 (4.8)	.58
Low birth weight [n = 43, (1/42) (n, %)]	0 (0)	5 (11.9)	.71

WD, Wilson’s disease.

**Table 5.** Pregnancy Outcomes According to Cirrhosis Status

	Cirrhotic Pregnancy (n = 68) (n, %)	Non-Cirrhotic Pregnancy (n = 63) (n, %)	*P*
Live birth (n, %)	46 (67.6)	52 (82.5)	.050
Spontaneous abortion (n, %)	20 (29.4)	10 (15.9)	.06
Stillbirth (n, %)	2 (2.9)	1 (2)	1.0
Premature birth [n = 53, (24/29) (n, %)]	4 (16.7)	5 (17.2)	1.0
Low birth weight [n = 61, (23/38) (n, %)]	5 (21.7)	1 (2.6)	.02

WD, Wilson’s disease.

## Data Availability

The data that support the findings of this study are available on request from the corresponding author.

## References

[1] Huster D. Wilson disease. Best Pract Res Clin Gastroenterol. 2010;24(5):531 539. (doi: 10.1016/j.bpg.2010.07.014) 20955957

[2] Poujois A Woimant F. Challenges in the diagnosis of Wilson disease. Ann Transl Med. 2019;7(Suppl 2):S67. (doi: 10.21037/atm.2019.02.10) PMC6531657 31179304

[3] Reuner U Dinger J. Pregnancy and Wilson disease: management and outcome of mother and newborns-experiences of a perinatal centre. Ann Transl Med. 2019;7(Suppl 2):S56. (doi: 10.21037/atm.2019.04.40) PMC6531655 31179293

[4] Grzeszczak K Kwiatkowski S Kosik-Bogacka D. The role of Fe, Zn, and Cu in pregnancy. Biomolecules. 2020;10(8):1176. (doi: 10.3390/biom10081176) PMC7463674 32806787

[5] Schilsky ML Roberts EA Bronstein JM A multidisciplinary approach to the diagnosis and management of Wilson disease: executive summary of the 2022 Practice Guidance on Wilson disease from the American Association for the Study of Liver Diseases. Hepatology. 2023;77(4):1428 1455. (doi: 10.1002/hep.32805) 36152019

[6] Pfeiffenberger J Beinhardt S Gotthardt DN Pregnancy in Wilson disease— management and outcome. Hepatology. 2018;67(4):1261 1269. (doi: 10.1002/hep.29490) 28859232

[7] Schilsky ML. Wilson disease diagnosis, treatment, and follow-up. Clin Liver Dis. 2017;21(4):755 767. (doi: 10.1016/j.cld.2017.06.011) 28987261

[8] Griebel CP Halvorsen J Golemon TB Day AA. Management of spontaneouseous abortion. Am Fam Physician. 2005;72(7):1243 1250. 16225027

[9] Tan J Surti B Saab S. Pregnancy and cirrhosis. Liver Transpl. 2008;14(8):1081 1091. (doi: 10.1002/lt.21572) 18668664

[10] Vishnupriya K Sheela CN Thayumanasundaram M. Maternal and perinatal outcome of Wilson disease in pregnancy: a 5-year Experience at a tertiary care center. J S Asian Fed Obstet Gynecol. 2017;9(4):318 322. (doi: 10.5005/jp-journals-10006-1521)

[11] Pinter R Hogge WA McPherson E. Infant with severe penicillamine embryopathy born to a woman with Wilson disease. Am J Med Genet A. 2004;128A(3):294 298. (doi: 10.1002/ajmg.a.10871) 15216551

[12] Brewer GJ Johnson VD Dick RD Hedera P Fink JK Kluin KJ. Treatment of Wilson’s disease with zinc. XVII: Treatment during pregnancy. Hepatology. 2000;31(2):364 370. (doi: 10.1002/hep.510310216) 10655259

[13] Litwin T Bembenek J Antos A The maternal and fetal outcomes of pregnancy in Wilson’s disease: a systematic literature review and meta-analysis. Biomedicines. 2022;10(9):2072. (doi: 10.3390/biomedicines10092072) PMC9495510 36140172

[14] Demirbas D Coelho AI Rubio-Gozalbo ME Berry GT. Hereditary galactosemia. Metabolism. 2018;83:188 196. (doi: 10.1016/j.metabol.2018.01.025) 29409891

[15] Brown AN Lange MM Aliasi-Sinai L Adverse pregnancy outcomes and effect of treatment in Wilson disease during pregnancy: systematic review and meta-analysis. Liver Int. 2024;44(11):3020 3030. (doi: 10.1111/liv.16072) 39206599

[16] Tarnacka B Rodo M Cichy S Członkowska A. Procreation ability in Wilson’s disease. Acta Neurol Scand. 2000;101(6):395 398. (doi: 10.1034/j.1600-0404.2000.90140a.x) 10877157

